# EEG Features in Young Patients with Syndromally Different Subtypes of Borderline Personality Disorder

**DOI:** 10.1192/j.eurpsy.2025.1921

**Published:** 2025-08-26

**Authors:** A. F. Iznak, A. A. Kuleshov, ?. V. Iznak, E. V. Damyanovich, V. G. Kaleda

**Affiliations:** 1 Laboratory of Neurophysiology; 2 Department of Youth Psychiatry, Mental Health Research Centre, Moscow, Russian Federation

## Abstract

**Introduction:**

The study of the neurobiological characteristics of borderline personality disorder (BPD) in youth is actual due to its high prevalence, but quantitative EEG studies of BPD have yielded mixed results.

**Objectives:**

The aim of the study was to assess the EEG features in patients with different clinical subtypes of borderline personality disorder (BPD).

**Methods:**

Total of 52 patients aged 16-25 years (mean age 20.4±3.2 years) with BPD (F60.31 by ICD-10) were enrolled in the study. Three groups of patients with different subtypes of BPD (with predominance of “affective storm”, “addictive adrenalin mania” and “cognitive dissociation”) were identified based on clinical and psychopathological characteristics. A pre-treatment multichannel resting EEG was recorded with measurements of EEG spectral power and coherence in narrow frequency sub-bands. Between-group differences in clinical and neurophysiological parameters were identified using Mann-Whitney criteria.

**Results:**

The groups did not differ in EEG spectral power values, but significant (p<0.05) differences between the groups were revealed in the spatial organization of the EEG namely in the number of “highly coherent” functional connections (with coherence coefficients above 0.9) that was the least in the group with “cognitive dissociation”. Low values of the number of such connections in the alpha2 EEG sub-band (9-11 Hz) in the frontal-central-temporal brain regions reflect a relatively poor functional state of the prefrontal cortex in this group.

**Conclusions:**

The noted features of the spatial functional organization of brain activity in patients with different BPD subtypes may underlie differences in their clinical conditions, in control of emotions and behavior.

**Disclosure of Interest:**

None Declared

